# Editorial: Carbon Catalysis: Focus on Sustainable Chemical Technology

**DOI:** 10.3389/fchem.2020.00308

**Published:** 2020-04-23

**Authors:** Zhigang Liu, Bingsen Zhang, Hao Yu, Kuang-Hsu Wu, Xing Huang

**Affiliations:** ^1^College of Chemistry and Chemical Engineering, Hunan University, Changsha, China; ^2^Shenyang National Laboratory for Materials Science, Institute of Metal Research, Chinese Academy of Sciences, Shenyang, China; ^3^School of Chemistry and Chemical Engineering, South China University of Technology, Guangzhou, China; ^4^School of Chemical Engineering, Faculty of Engineering, University of New South Wales, Sydney, NSW, Australia; ^5^ETH Zürich, Zurich, Switzerland

**Keywords:** photocatalyst, metal-organic frameworks, carbon quantum dot, carbon supported catalyst, single atom catalyst, carbon-based electrocatalysts

Carbon materials are widely applied as a key ingredient of industrial catalysts. Compared to transition metal oxides, carbon can form different architectures as an inert support, which is popularly used as a platform to study the surface chemistry and explore the unique changes of electronic state of metal catalysts (Su et al., 2013; Zhang and Su, 2015). For instance, carbon nanotubes (CNTs) are widely used in heterogeneous catalysis due to their chemical stability, relatively low cost and easy recovery of precious metals by means of combustion in recent years (Gao et al., 2018; Zhu et al., 2019). CNTs have been demonstrated effective to support noble metal nanoparticles to catalyze the valorization of biomass-derived platform molecules in the context of sustainable chemistry (Wan et al., 2014; Ning et al., 2015, 2016; Meng et al., 2019). However, the separation of CNT-based catalyst usually adds costs particularly for liquid-phase reactions due to its original powder form. As an ingenious solution, shaping the catalyst into a CNT-based monolith would facilitate separating and handling catalysts, even enable the fabrication of multifunctional reactors for process intensification, which thus present a great potential for industrial liquid/gas phase heterogeneous catalysis (Mu et al., 2016; Ba et al., 2017; García-Bordejé et al., 2017).

Beyond a supporting material, nanocarbon materials have demonstrated promising features for metal-free catalysis including selective oxidation, hydrogenation, dehydrogenation, oxygen reduction electrocatalysis, water splitting and CO_2_ reduction reaction (CO2RR), and so on. Owing to their advantages, such as large surface areas, high adsorption capacity, excellent thermal and mechanical stability, outstanding electronic properties, tunable porosity and surface chemistry, nanocarbons are of high capability to anchor and disperse the active metal nanoparticles to exhibit versatile functions (Su et al., 2013). To-date, there has been significant effort on this topic to discover the beautiful chemistry behind nanocarbon materials.

To unravel the unprecedented performance comparable to noble metal catalysts on carbon-based catalysts, transition metals (Co, Fe, Ni) and nitrogen co-doped carbon nanomaterials (M-N-C) have been used in the hydrogenation and oxidation reactions (Cheng et al., 2015; Ao et al., 2019; Huang et al., 2019; Liu et al., 2019). For instance, Co-N-C catalysts have been investigated in selective oxidation of aromatic hydrocarbons and exhibited attractive catalytic performance (Jie et al., 2017).

Carbon quantum dots (CQDs) (Semeniuk et al., 2019) are novel zero-dimensional carbon-based nanomaterials known for their quantum-size effect and the relatively strong fluorescence characteristics. Graphene quantum dots (GQDs), carbon nanodots (CNDs), and polymer dots (PDs) are key members in this family (Zhu et al., 2015). Especially, CQDs have been widely applied in several electrocatalytic reactions, including oxygen reduction reaction (ORR), oxygen evolution reaction (OER), hydrogen evolution reduction (HER) and CO2RR.

To solve the energy crisis and environmental pollution, photocatalysts have been playing a key role to reduce toxic contaminants and produce solar H_2_ (Hisatomi et al., 2014; Dai et al., 2017). However, challenges for photocatalysts reside at the limited light absorption, high charge recombination and low quantum yields (Sudhaik et al., 2018). While various photocatalysts have been developed to date, carbon-based photocatalysts have aroused tremendous interests due to their versatile chemistry and non-toxicity to the environment (Xia et al., 2017). These unique properties make carbon nanomaterials one of the most promising candidates as photocatalysts (Yu et al., 2014).

Metal-organic frameworks (MOFs) are porous crystalline materials formed via self-assembly of metal ions and organic ligands (Li et al., 2019), which may provide an preferential structural platform to get novel metal-carbon hybrid electrocatalysts with unique properties such as chemical composition versatility, high porosity and high surface areas (Ma et al., 2014). However, owing to the poor thermal stability of single metal nodes, MOF-derived carbons usually face the problem of a significantly reduced specific surface area after usage, which may limit their applications (Shui et al., 2015). Recently, it was reported that partial replacement of metal ions in the MOF framework with a second metal with similar properties can not only keep their original structure but also endow more functionalities (Wang et al., 2016). As a result, it is a promising strategy to design bi-/multi-metallic MOF derivatives with favorable chemical compositions and desirable structure for developing cost-effective noble-metal-free electrocatalysts that can present a catalytic performance as well as, or even better than noble metal catalysts.

At the same time, numerous works have been made to develop low-cost earth-abundant electrocatalysts, such as the carbon-based materials and transition metal sulfides and carbides (Feng et al., 2015). Among these electrocatalysts, carbon-based materials, including heteroatom-doped carbons and carbon-encapsulated metals, have also attracted increasing attentions for their low-cost, high-efficiency, and good long-term durability.

In this Special Issue, we present some selected examples to illustrate the use of these carbon-based materials for catalysis as an inspiration and stimulation to the community. We expect more and more interests from researchers of various disciplines and conduct cutting-edge works to advance the knowledge and application relevant to carbon-based materials, given the many wonderful merits of this black gold. As Guest Editors of this Research Topic, we would like to thank all the authors for their valuable contributions to this special issue. We also thankfully acknowledge the referees and the editors of *Frontiers in Chemistry*.

## Author Contributions

All authors listed have made a substantial, direct and intellectual contribution to the work, and approved it for publication.

## Conflict of Interest

The authors declare that the research was conducted in the absence of any commercial or financial relationships that could be construed as a potential conflict of interest.
